# PAIN, PAIN MANAGEMENT, AND CONSEQUENCES OF PAIN AMONG AL RESIDENTS

**DOI:** 10.1093/geroni/igz038.880

**Published:** 2019-11-08

**Authors:** Barbara Resnick

**Affiliations:** University of Maryland School of Nursing, Baltimore, Maryland, United States

## Abstract

The purpose of this study was to describe the incidence, management and impact of pain on function, agitation, and resistance to care. This was a descriptive study using baseline data from 260 residents in the second cohort of the FFC-AL-EIT study. The majority of the sample was female (71%) and white (96%) with a mean age of 87 (SD=7). Fifty-two residents (20%) reported pain based on objective (PAINAD) or subjective (verbal descriptive scale, VDS) pain assessments. A total of 75 residents (29%) received pain medication and 22 (42%) individuals reporting pain were not getting pain medication. Controlling for age, gender and cognition, PAINAD was significantly associated with agitation, function, and resistance to care and the VDS was only associated with function. Pain assessments should include objective and subjective measures and management of pain should be considered as it may help to optimize function and decrease behavioral symptoms among AL residents.

